# Basic surface-active properties in the homologous series of β-alkyl (C_12_H_25_/C_18_H_37_) polyethyleneoxy (n = 0-20) propionamides

**DOI:** 10.1186/1752-153X-7-31

**Published:** 2013-02-13

**Authors:** Adrian Riviş, Gabriel Bujanca, Teodor Traşcă

**Affiliations:** 1 Department of Food Science, Faculty of Food Processing Technology, Banat’s University of Agricultural Sciences and Veterinary Medicine, Calea Aradului 119, Timişoara, 300645, Romania

**Keywords:** β-substituted higher propionamides, Polyethoxylated amides, Colloidal characteristics, Higher aliphatic amides, Hydrophilic-hydrophobic balance, Critical micelle concentration

## Abstract

**Background:**

Heterogeneous β-Alkyl (C_12_H_25_/C_18_H_37_) polyethyleneoxy (n = 0-20) propionamides [R(EO)_n_PD] represent new “hybrid” nonionic-ionic colloidal structures in the field of surface-active products (technical products). These “niche” compounds have three structural and compositional characteristics that also define their basic colloidal properties: mixture of R and PEO chain homologues; specific conformations due to the PEO chains; and the presence of side products from the addition of higher alcohols, polyethyleneglycols and traces of water to acrylamide. The proposed major objective of this paper is the basic informative colloidal characterization (functional classification, HLB balance, surface tension, critical micelle concentration) in direct correlation with the structural changes in the homologous series of LM(EO)_n_PD and CS(EO)_n_PD. The structures were obtained either indirectly by cyanoethylation followed by partial acid hydrolysis of the corresponding β-propionitriles, or directly by the nucleophilic addition under alkaline catalysis of linear higher alcohols C_12_H_25_/C_14_H_29_ (7/3) (LM) and C_16_H_33_/C_18_H_37_ (CS) as such and heterogeneous polyethoxylated (n = 3-20) to acrylamide monomer, through an adapted classic reaction scheme.

**Results:**

In the series of basic colloidal characteristics investigated the structure-surface activity dependence is confirmed. Their indicative character for R(EO)_n_PD is based on the assumption that the structures studied are not unitary (heterogeneous) because: a) the hydrophobic chains C_12_H_25_/C_18_H_37_ have been grouped in two variants, C_12_H_25_/C_14_H_29_ (LM); C_16_H_33_/C_18_H_37_ (CS), each with an internal mass ratio of 7/3; b) the hydrophilic polyoxyethylene chains (n = 3-20) have polydisperse character; the meaning and value the oligomerization degree, n, is that of weighted average. In these conditions the surface tension increases proportionally with the oligomerization degree of the polyoxyethylene chain, while the critical micelle concentration decreases in the same homologous series as well as with the increase of the hydrophobic chain in the C_12_H_25_ to C_18_H_37_ series. A mechanism of micellization is proposed, consistent with the experimental data recorded and the hypotheses known from the consulted literature.

**Conclusions:**

The idea of the obtaining and basic colloidal characterization of heterogeneous R(EO)_n_PD is justified. The knowledge and constructive approach of the heterogeneous character confirm the basic surface-active potential of R(EO)_n_PD, the structure-colloidal characteristics dependence and justifies further, more extensive research.

## Background

Higher primary, secondary and tertiary aliphatic amides, known for their low solubility in aqueous floats are valued sanitation components (“cosurfactants”) by association in various anionic, anionic-nonionic, etc., surface-active colloidal systems as such or salified as formates and acetates, respectively [1-5].

Heterogeneous R(EO)_n_PD represent a relatively small new range of surface-active compounds with still limited audience due to their recent reporting [6] in the market of surface-active compounds and the absence of research in the field, marked by three distinct aspects:

✓ the heterogeneous character of the hydrophilic polyoxyethylene chain (PEO) (the statistical dispersion of the oligomerization degree, n) [2,3,7,8] and of the hydrophobic chain (R = C_12_H_25_ to C_18_H_37_), respectively;

✓ specific conformational characteristics of the PEO chains depending on the size of the oligomerization degree, n [8-14];

✓ the possible and very probable presence of some byproducts characteristic of the industrial synthesis of polyethoxylated higher alcohols, R(EO)_n_H (free higher alcohols, ROH, free polyethyleneglycols, PEG_n_ and traces of water of hygroscopicity) [3,8,15].

The study of the informative basic colloidal characteristics of R(EO)_n_PD as such has recently come to our attention with the intention to associate these surface-active structures in perspective as cosurfactants in future sanitation recipes in the CIP (“clean in place”) system, together with nonionic soaps belonging to the same structural family [alkaline and ammonium β-alkyl C_12_H_25_/C_14_H_29_ (7/3) (LM) and C_16_H_33_/C_18_H_37_ (7/3) (CS) polyethyleneoxy (n = 0-20) propionates] R(EO)_n_PC^-^[16-20].

Research similar to that presented in this paper has not been reported in the published and consulted literature in the last seven decades, although after 1977 [21-23] the interest for PEGylation as an inner PEG_n_ attachment process is constantly increasing.

PEGylation involves the successive covalent grafting of new structures at the two terminals (free hydroxyl functional groups) of polyethyleneglycols. The newly formed covalent bond can temporarily “mask” the “active vector” support (host).

The polyethoxylated higher alcohols (C_8_H_17_ - C_18_H_37_) can be considered as monoderivatized polyethyleneglycols (primary PEGylation fragment). Obtained industrially by the reaction of linear or branched higher alcohols with ethylene oxide under an inert atmosphere, basic catalysis at 90-150°C, they are characterized as a mixture of polyoxyethylene chain homologues with statistically distributed molecular weight, along with varying amounts of free higher alcohols, free polyethyleneglycols and traces of water (Table 1) [8].

**Table 1:** **The main physico-chemical characteristics of higher alcohols (ROH) (mixture of homologues) and of heterogeneous polyethoxylated higher alcohols R-(EO)**_**n**_**-H**

**Product name**	**Hydrocarbon chain (R)**	**Notation**	**Appearance**	**Hydrocarbon chain distribution (%)**^**2)**^				**Physico-chemical characteristics**			
				**C**_**12**_	**C**_**14**_	**C**_**16**_	**C**_**18**_	**Polyethyleneglycols content PEG (%)**	**Higher alcohols (%)**	**Polyethoxylated higher alcohols, mixtures of chain homologues (%)**	**H**_**2**_**O (%)**
Lauryl/myristyl alcohol	C_12_H_25_/C_14_H_29_	LM-O-H	colorless fluid	70	30	-	-	-	99.55	-	-
Cetyl/stearyl alcohol	C_16_H_33_/C_18_H_37_	CS-O-H	waxy colorless solid	-	-	70	30	-	99.24	-	-
Polyethoxylated (n = 3) lauryl/myristyl alcohol	C_12_H_25_/C_14_H_29_	LM-(EO)_3_-H	colorless fluid	70	30	-	-	0.43 (−)^1)^	15.03 (−)^1)^	84.24 (99.12)^1)^	0.30 (−)^1)^
Polyethoxylated (n = 3) cetyl/stearyl alcohol	C_16_H_33_/C_18_H_37_	CS-(EO)_3_-H	colorless solid	-	-	70	30	0.52	17.36	82.78	0.32
Polyethoxylated (n = 6) lauryl/myristyl alcohol	C_12_H_25_/C_14_H_29_	LM-(EO)_6_-H	viscous colorless fluid	70	30	-	-	0.61	4.58	88.36	0.58
Polyethoxylated (n = 6) cetyl/stearyl alcohol	C_16_H_33_/C_18_H_37_	CS-(EO)_6_-H	waxy colorless solid	-	-	70	30	0.74	5.18	85.15	0.49
Polyethoxylated (n = 9) lauryl/myristyl alcohol	C_12_H_25_/C_14_H_29_	LM-(EO)_9_-H	colorless paste	70	30	-	-	0.78	0.88	92.45	0.68
Polyethoxylated (n = 9) cetyl/stearyl alcohol	C_16_H_33_/C_18_H_37_	CS-(EO)_9_-H	waxy colorless solid	-	-	70	30	0.93	1.25	89.63	0.88
Polyethoxylated (n = 12) lauryl/myristyl alcohol	C_12_H_25_/C_14_H_29_	LM-(EO)_12_-H	waxy colorless solid	70	30	-	-	0.95	0.25	97.48	0.90
Polyethoxylated (n = 12) cetyl/stearyl alcohol	C_16_H_33_/C_18_H_37_	CS-(EO)_12_-H	waxy colorless solid	-	-	70	30	1.18	0.98	93.47	1.02
Polyethoxylated (n = 16) lauryl/myristyl alcohol	C_12_H_25_/C_14_H_29_	LM-(EO)_16_-H	waxy colorless solid	70	30	-	-	1.19	-	98.52	1.15
Polyethoxylated (n = 16) cetyl/stearyl alcohol	C_16_H_33_/C_18_H_37_	CS-(EO)_16_-H	waxy colorless solid	-	-	70	30	1.47	0.73	96.51	1.42
Polyethoxylated (n = 20) lauryl/myristyl alcohol	C_12_H_25_/C_14_H_29_	LM-(EO)_20_-H	waxy colorless solid	70	30	-	-	1.79	-	98.11	1.58
Polyethoxylated (n = 20) cetyl/stearyl alcohol	C_16_H_33_/C_18_H_37_	CS-(EO)_20_-H	waxy colorless solid	-	-	70	30	1.93	0.46	98.23	1.74

^1)^ values in parentheses refer to “homogeneous” polyethoxylated (n = 3) lauryl/myristyl (7/3) alcohol.

^2)^ determined by gas chromatography [15,16].

The increase of the average polyethoxylation degree, n, result in the widening of the spectrum of PEO chain homologues [8]. Separation into unitary structures through physico-chemical methods (molecular distillation, liquid-liquid extraction, column chromatography, etc.) is difficult [16], but the synthesis of the “homogeneous” polyoxyethylene PEO chain becomes possible through several “step by step” versions [8]: condensations with monohalogeno-glycols [24]; catalytic reduction of esters [25]; the Williamson ether synthesis [26]; etherification of higher n-alkyl tosylates (C_8_H_17_ - C_18_H_37_) [27].

The Williamson scheme of synthesis of “homogeneous” highly polyethoxylated higher alcohols (C_8_H_17_ - C_18_H_37_), known as two variants, with all the difficulties of synthesis, purification, separation, remains the most widely used method by:

✓ successive attachment lower oxyethylene units (n = 2-3) to a “homogeneous” hydrocarbon chain [28];

✓ purification and subsequent attachment of oxyethylene units (n ≥ 3) to the “homogeneous” hydrocarbon chain [29].

The second variant, characterized by yields not exceeding 60%, laborious and inefficient separation of the higher alcohols is less recommended [8].

The chemistry and applications of derivatized polyoxyethylene chains (PEO) as such or condensed with polyoxypropylene chains (PPO) led to biocompatible structures extensively studied and covered in the literature over the past four decades [23,30].

After the reporting of proteins covalently grafted with PEG_n_ (conjugates) [21,22] and the pioneering attempts of (Davis F. et al. 1979), their potential in the conditioning of active vectors in the most diverse areas was recognized. PEGylation as an experimental technique benefits from a wide coverage, at the same time with the chemistry, analytical methods and technical applications that have become more and more elaborate.

The favorable endorsement (after 1990) of the new PEG_n_ conjugates by the FDA in the U.S.A. confirmed the maturity of these products and technologies.

New PEG_n_ architectures justify the research efforts in mono- or bis-derivatization of PEO chains.

Are accepted with convincing arguments [23,30] the similarities between the primary, secondary and tertiary structure of the macromolecular biochains in polyethyleneglycols, as such and derivatized, compared to proteins, lipids and polysaccharides in the active and passive transfer processes of material biocarriers of utilities.

Also “highly qualified” (“specialized”) structures have been designed and built, in the range of PEG_n_-L (vegetal lipids) conjugates, employing processing and evaluation block schemes based on the insertion of PEO chains in the most various areas of activity.

**Figure 1:**
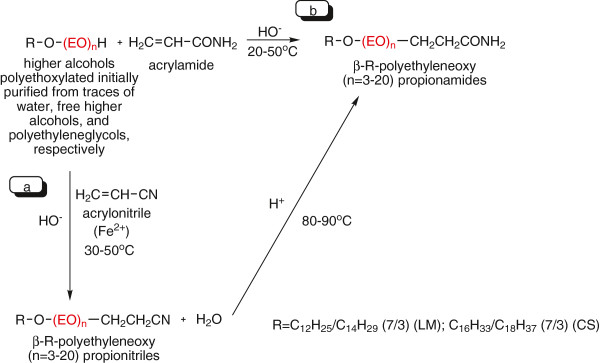
**The reaction scheme for the preparation of C**_**12**_**H**_**25**_**/C**_**14**_**H**_**29 **_**(7/3) C**_**16**_**H**_**33**_**/C**_**18**_**H**_**37 **_**(7/3) polyethyleneoxy (n = 3-20) propionamides.**

Therefore the paper sets as its major objectives the indicative basic colloidal characterization (functional classification, surface tension, critical micelle concentration, hydrophilic-hydrophobic balance) in the heterogeneous homologous series of LM and CS, respectively, polyethyleneoxy (n = 0-20) propionamides (Figure 1), obtained by a classic reaction scheme [15-17,19,20]:

✓ indirectly by the nucleophilic addition of higher alcohols, as such and polyethoxylated (n = 0-20), initially purified from traces of water, free higher alcohols, and polyethyleneglycols, respectively, to acrylonitrile, followed by the partial acid hydrolysis of β-substituted propionitriles to β-substituted propionamides (Figure 1a);

✓ directly by the nucleophilic addition of higher alcohols, as such and polyethoxylated (n = 0-20), initially purified from traces of water, free higher alcohols, and polyethyleneglycols, respectively, to acrylamide under basic catalysis (Figure 1b).

## Results and discussions

The main colloidal characteristics of the surface-active compounds (technical products) depend on their structure and heterogeneous composition. R(EO)_n_PD as mixtures with widespread distribution of hydrophobic, R, and hydrophilic PEO, respectively, chain homologues, manifest themselves cumulatively through the individual colloidal behavior of the present unitary structure and through mutual interdependencies. Therefore the experimental values of the basic main colloidal characteristics, evaluated in such a casuistry, also have an indicative character even if they are the result of the mathematical processing of a considerable number of measurements. The selection and use of the most appropriate methods of surface-active evaluation can also generate comments for and against, justified both by the fidelity of the recording and interpretation of the colloidal phenomenon, but sometimes mostly by the absence of universally accepted operating protocols. In this work standardized methods are preferred, nationally approved and recognized in Europe or completed with ISO recommendations if the case. Laboratory evaluations performed on the homologous series (n = 0-20) of the two sets of β-substituted higher aliphatic propionamides purified prior to the synthesis (Figure 1) fall into this casuistry.

Mathematical processing of the indicative basic colloidal characteristics of the structures of the homologous series studied allowed the formulation of structure-surface activity dependencies (empirical mathematical relationships, most of them with correlation coefficients between 0.9 and 1) and the most probable mechanisms of surface activity.

### Functional classification

To assess and explain why LM(EO)_n_PD and CS(EO)_n_PD possess surface-active properties, some general considerations on their amphiphilic molecular structure are necessary.

Synergistic cumulation in the same surface-active structural architecture of the amide functional group with one or more polyoxyethylene chains (hydrophilic) with different oligomerization degrees (n) led to the first polyethoxylated amides. The structures represent the first higher aliphatic amides with a polyoxyethylene chain inserted between a determinant hydrophobic group and a polar nonionic hydrophilic group (the primary amide function).

**Figure 2:**
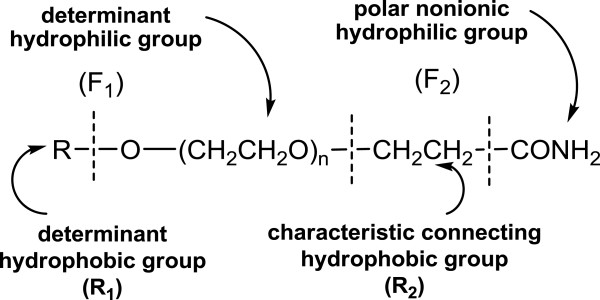
**The network of the heterogeneous R(EO)**_**n**_**PD homologous series.**

Due to their composition, specific primary and secondary (conformational) structure, R(EO)_n_PD have a pronounced heterogeneous character. The idea of their basic colloidal characterization becomes indicative, being the resultant of the colloidal manifestation of 24 unitary homologous structures (4 hydrophobic R series, each with 6 hydrophilic PEO series) (Figure 2). By the homogeneous R(EO)_n_PD structure in the sense of this work we mean a strictly unitary β-substituted polyethoxylated higher aliphatic propionamide (Figure 2). Similarly 23 more unitary homologous (“homogeneous”) structures can be mentioned. Exhaustive isolation into individual (unitary) structures, laborious, conceptually bold, but difficult to accomplish, did not constitute a major objective of the paper also due to the pronounced manifestation of the “neighboring effects” (“sympathy effects”) between two or more adjacent chain homologues [16,17], with similar physico-chemical constants that do not allow a certain exhaustive separation.

**Figure 3:**
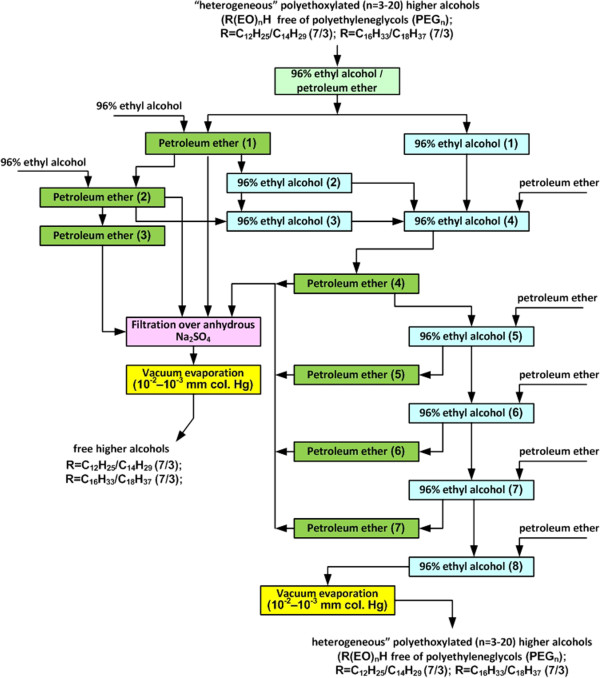
**Block diagram of operations of the process of elimination by liquid-liquid extraction of polyethyleneglycols (PEG**_**n**_**) from the mixture of dehydrated “heterogeneous” polyethoxylated (n = 3-20) higher alcohols R(EO)**_**n**_**H (adapted from Schick, M., 1987) [**[8]**].**

**Figure 4:**
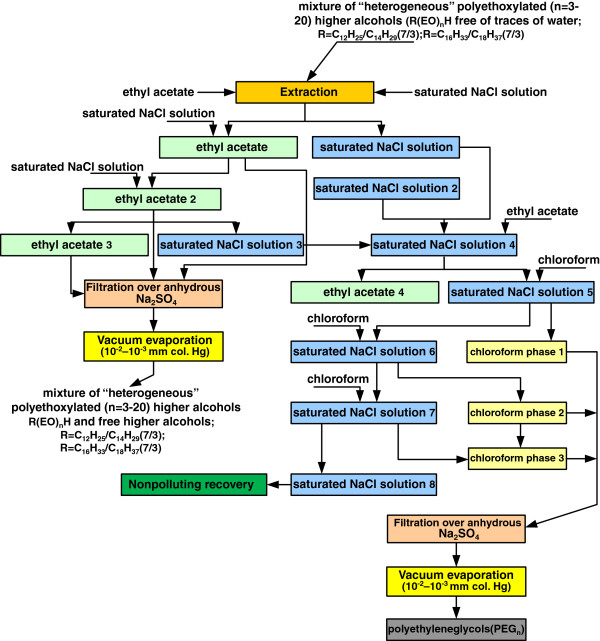
**Functional structure of β-alkyl (R = C**_**12**_**H**_**25 **_**to C**_**18**_**H**_**37**_**) polyethyleneoxy (n = 3-20) propionamides [R(EO)**_**n**_**PD] [**[31]**].**

Of the three issues mentioned in the background, only the influence of secondary products was eliminated *a priori* by repeated liquid/liquid extractions in appropriate solvent systems (Figures 3, 4). The other two variables in the system can be eliminated simultaneously, if the heterogeneous structural character of the R and PEO chains is removed through a directed “step by step” Williamson-type synthesis [16,17].

**Figure 5:**
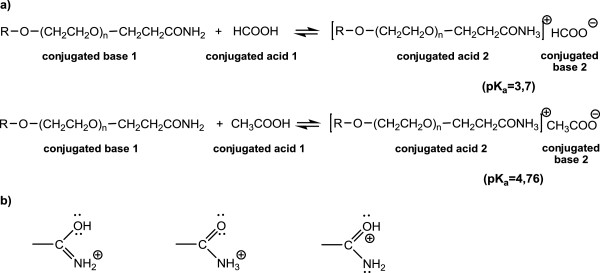
**Block diagram of operations of the process of purification by liquid-liquid extraction (petroleum ether, b.p. = 30-60°C/ethyl alcohol 96%) of “heterogeneous” polyethoxylated (n = 3-20) higher alcohols R(EO)**_**n**_**H, of free higher alcohols (ROH) (adapted from Schick, M., 1987) [**[8]**].**

**Figure 6:**
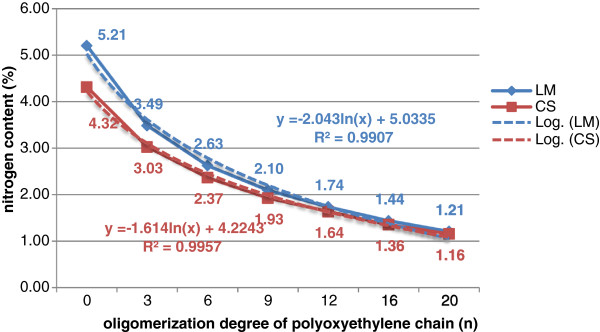
**Acid–base equilibrium in the “salification” process of R(EO)**_**n**_**PD with formic and acetic acid (a) and electron mobility in the primary amide functional group (b).**

The inclusion of β-alkyl (C_12_H_25_/C_18_H_37_) polyethyleneoxy (n = 3-20) in the category of surface-active agents involves the nomination of the hydrophilic (lipophobic)/hydrophobic (lipophilic), polar/non-polar (amphiphilic) domain, which determines their colloidal character. The many possibilities for modifying the determinant hydrophobic fragment between the C_12_H_25_/C_14_H_29_ and C_16_H_33_/C_18_H_37_ structural variants require additional information (the mutual interchain mass ratio of 7/3). The determinant hydrophilic fragment, although relatively simple structurally (polyether chain), also requires additional structural specifications with respect to the conformation and the size of the oligomerization degree (n) of the PEO chain, respectively (Figure 5). The nonionic hydrophilic primary amide functional group grafted at end of β-alkyl substituted polyethyleneoxy propionamides presents low basicity and polarity due to antagonistic electromeric effects in its structure [6]. The most obvious confirmation is that the structures salified as formates (HCOO^-^) and acetates (CH_3_COO^-^), respectively, can be theoretically considered “salts”, even if they are extremely unstable and decompose in aqueous solutions with recovery of primary amides. If we interpret these developments in terms of acid–base equilibrium and the pK_a_ values of the two conjugate structures (Figure 6), we are convinced of these realities [32].

Also important for the colloidal behavior of R(EO)_n_PD is the potential interaction between the two polar hydrophilic structural fragments: the primary amide group and the PEO chain. The former contains three elements from the second row of the periodic table with increasing electronegativity C (IV) < N (V) < O (VI). The presence and involvement of non-bonding electrons of nitrogen and oxygen in the p-π conjugation has major consequences on the properties of the function as a whole: low basicity (K_b_ ca. 10^-14^); ability to form hydrogen bonds; amidic conjugation; the predominantly flat conformation of the partially double carbon-nitrogen bond with limitation of free coaxial rotation and tautomerism (equilibrium between amide and iminol forms) [6,33]. All these support the flexible cationic character of the functional group as a whole [32,33] which can interact with the PEO conformation of different sizes.

**Figure 7:**
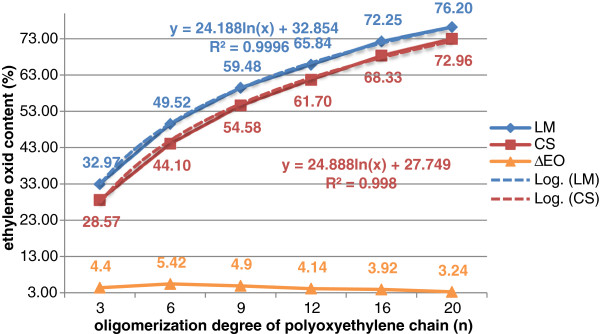
**Dependence of the nitrogen content (%) on the oligomerization degree (n) of the polyoxyethylene chain in the homologous series of LM(EO)**_**n**_**PD and CS(EO)**_**n**_**PD, respectively.**

From the comparative analysis of the elemental composition it is found that the logarithmic dependence equation in Figure 7 with a correlation coefficient R^2^>0.99, confirms the reduction of the proportion of amide nitrogen in both homologous series simultaneously with the increase of the oligomerization degree (n) of the polyoxyethylene chain in the structure. Up to the corresponding value (n ≤ 12) of the homologous series the nitrogen content is higher for the LM chain, then for n ≥ 12 it is very similar, virtually identical for both homologous series. Therefore simultaneously with the increase of the weight of the polyoxyethylene chains in the architecture of β-substituted aliphatic propionamides studied, the weight and influence of the primary amide group, already low as nonionic hydrophilic group, decreases even further.

With reduced polarity (basicity) and decreased influence on the whole structure, they still form inter- and intramolecular hydrogen bonds, keeping the conformation planar due mainly to the p-π conjugation in the structure.

The second major structural unit of the β-substituted propionamides studied is represented by the polyoxyethylene chains, determinant hydrophilic fragment with increasing oligomerization degree.

**Figure 8:**
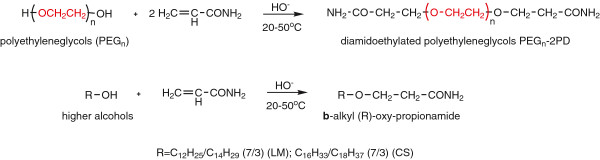
**Dependence of the ethylene oxide content (%)on the oligomerization degree (n) of the polyoxyethylene chain in the homologous series of LM(EO)**_**n**_**PD and CS(EO)**_**n**_**PD, respectively.**

Their properties influence the overall colloidal behavior of LM(EO)_n_PD and CS(EO)_n_PD, the mechanisms of action (orientation effects at the interface, mutual association affinities, development of the hydrophilic character, etc.). The dependence between the ethene oxide content and the oligomerization degree (n) of the polyoxyethylene chain (Figure 8) in the homologous series studied follows a logarithmic mathematical relationship with a correlation coefficient R^2^>0.99. Between the two dependence curves referring to the LM and CS hydrophobic chains, the difference in the ethylene oxide content (ΔEO) determined experimentally (average value), ΔEO = 4.337, suggests a close evolution between it and the calculated value (Table 2). The explanation is that the polyethoxylated higher alcohols (technical products) employed initially as raw materials were purified exhaustively of free higher alcohols and polyethyleneglycols.

**Table 2:** **The main physico-chemical characteristics of LM(EO)**_**n**_**PD and CS(EO)**_**n**_**PD**

**No.**	**Product**	**Nitrogen content (%)**		**Ethylene oxide content (%)**	
		**Determined**^**1**^	**Calculated**	**Determined**^**1**^	**Calculated**
1.	β-lauryl/myristyl (7/3) oxy-propionamide	5.21	5.26	-	-
2.	β-cetyl/stearyl (7/3) oxy-propionamide	4.32	4.36	-	-
3.	β-lauryl/myristyl (7/3) polyethyleneoxy (n = 3) propionamide	3.50	3.52	32.97	33.22
4.	β-cetyl/stearyl (7/3) polyethyleneoxy (n = 3) propionamide	3.03	3.09	28.57	29.11
5.	β-lauryl/myristyl (7/3) polyethyleneoxy (n = 6) propionamide	2.63	2.64	49.53	49.87
6.	β-cetyl/stearyl (7/3) polyethyleneoxy (n = 6) propionamide	2.37	2.39	44.78	45.10
7.	β-lauryl/myristyl (7/3) polyethyleneoxy (n = 9) propionamide	2.10	2.12	59.48	59.87
8.	β-cetyl/stearyl (7/3) polyethyleneoxy (n = 9) propionamide	1.93	1.95	54.58	55.20
9.	β-lauryl/myristyl (7/3) polyethyleneoxy (n = 12) propionamide	1.75	1.76	65.84	66.55
10.	β-cetyl/stearyl (7/3) polyethyleneoxy (n = 12) propionamide	1.64	1.65	61.70	62.16
11.	β-lauryl/myristyl (7/3) polyethyleneoxy (n = 16) propionamide	1.44	1.44	72.25	72.62
12.	β-cetyl/stearyl (7/3) polyethyleneoxy (n = 16) propionamide	1.36	1.36	68.33	68.66
13.	β-lauryl/myristyl (7/3) polyethyleneoxy (n = 20) propionamide	1.21	1.22	76.20	76.83
14.	β-cetyl/stearyl (7/3) polyethyleneoxy (n = 20) propionamide	1.16	1.17	72.96	73.25

^1^ Kjeldahl method; ^2^ cleavage with concd. HI (d = 1.7 – 1.9).

Changes in the various structural units present in surfactants strongly affect the interfacial properties. Such properties as surface tension reduction, micelle formation etc. show marked changes with variations in both the hydrophilic and hydrophobic portions of the surfactant molecule, reflecting the processes occurring on molecular level. Changes in these properties caused by such factors as the length and nature of hydrophobic group, the nature of the hydrophilic group and its position in the molecule, and the presence or absence of an ionic charge are described and explained in terms of the molecular processes involved [34].

### Physico-chemical characterization of R(EO)_n_PD

Optimizing the solubility and hence their basic colloidal characteristics has been a constant concern of researchers in this area. “Salification” as formates or acetates practically limited accessing higher aliphatic amides only to the acid range of the sanitation protocols.

The water solubility of EO fatty amides derivatives essentially depends on both the temperature and HLB, which can be easily calculated indicatively according to Griffin’s equation: HLB = E/5; where E is the weight percentage of ethylene oxide units in the considered molecule.

A simple approximation of water solubility under current conditions can be made by considering the ratio of ethylene oxide units (EO) to the carbon number (N). If this ratio EO/N is below to 1/2, solubility is barely achieved; if this ratio EO/N is equal to 1/2, solubility is fairly good; if this ratio EO/N is equal to 3/2, solubility is very high. The maximum surface activity of nonionics is observed near the “cloud point” [1-4].

The products of the studied homologous series are alkylpolyethyleneglycol ethers, heterogeneously derivatized at the second end with the propionamide fragment. Characterized primarily physico-chemically (appearance, viscosity, water solubility), the “parent compound” (n = 0) of both homologous series of hydrocarbon chains (LM and CS) stands out from the rest of representatives, being solid, waxy white-gray (20°C), insoluble in cold and hot (60°C) water, where even in dilute solutions form opalescent, inhomogeneous multiphase systems. From these considerations it cannot be considered properly speaking surface active.

Following the homologous series both for the determinant hydrophobic fragment, LM and CS, and for the determinant hydrophilic one, (EO)_n_ (n = 3-20), respectively, viscosity increases, and the white-gray color in the solid state becomes constantly yellow-brownish in the melt (fluid state), while the solubility and appearance of the aqueous solution evolves from homogeneous opalescent to clear homogeneous. These considerations are found for the homologous series of LM(EO)_n_PD and CS(EO)_n_PD, respectively, synthesized from the corresponding polyethoxylated higher alcohols, previously purified from free higher alcohols (ROH), free polyethyleneglycols (PEG)_n_ and traces of water. When employing for the study the homologous series of unpurified heterogeneous R(EO)_n_PD [16,18,20], the experimental data recorded were significantly modified, because these contained: decreasing amounts of R-O-PD, in the 17.3% - 0.46% range; polyethyleneglycols bis-derivatized with propionamide fragments between 0.56% - 1.93%, both components with surface-active properties, able to form mixed micellar architectures similar to those presented below.

### Composition and structural characteristics of R(EO)_n_PD

The industrial alkylation of higher alcohols with ethylene oxide (EO) is a method widely used in obtaining polyethoxylated higher alcohols, R(EO)_n_H. Inevitably a technical product is obtained, where can be found in varying proportions (Table 1), besides R(EO)_n_H themselves (mixture with a statistical distribution of polyoxyethylene chain homologues), polyethylenglycols (PEG_n_), free higher alcohols (R-OH) and traces of hygroscopically retained water [3,4,7,8].

**Figure 9:**
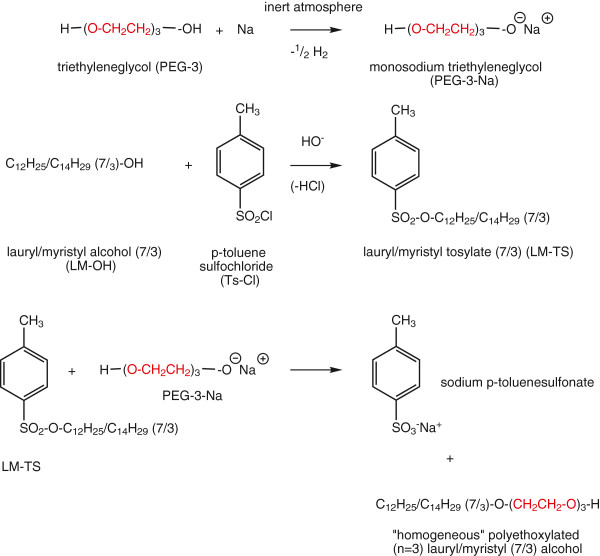
**Scheme of the nucleophilic addition of PEG**_**n **_**and R-OH (side products) in R(EO)**_**n**_**H (technical products) to acrylamide.**

These are bearing hydroxyl groups, able to actively participate through nucleophilic addition to the activated double bond (−I_s_; -E_s_) in acrylonitrile and acrylamide, with the formation of products that influence determinantly the colloidal behavior of R(EO)_n_PD (Figure 9).

From the analysis of the experimental values (Table 1) on the “heterogeneous” higher polyethoxylated alcohols, raw materials in the synthesis of R(EO)_n_PD, the following aspects with consequences on their subsequent behavior can be observed:

✓ the share of the free higher alcohols (ROH) in technical products is high at the beginning of the homologous series (n = 3) and superior for the CS homologous series as against the LM one, but decreases with increasing the size of the PEO chain up to complete for elimination the R(EO)_n_H series (n = 16;20);

✓ the share of the free polyethyleneglycols (PEG_n_) increases in both homologous series of hydrophobic chain simultaneously with the increase of the oligomerization degree (n), more so in the CS series as against the LM one;

✓ the water content (pronounced hygroscopicity of all representatives of the homologous series) increases with the size of the PEO chain, more pronouncedly for the CS series as against the LM one;

✓ the share of the mixture of PEO chain homologues for the polyethoxylated higher alcohols themselves, lower for n = 3 (81-84%) exceeds 93% for the rest of the homologous series (n = 6, 9, 12, 16, 20);

✓ gas-chromatographic analysis of the purified polyethoxylated higher alcohols R(EO)_n_H or the nucleophilic addition products R(EO)_n_PN, R(EO)_n_PD, confirms the broad statistical distribution of the PEO chain homologues (for the purified technical product LM(EO)_3_PN n = 3, the range of PEO chain homologues includes the interval n = 1-5, doubles if the hydrophobic L/M chain homologues are also considered [15,16,35].

From Table 1 it may be noted that in the heterogeneous alcohol LM(EO)_3_H are present all the side products previously specified in the paper.

After purification of free alcohol LM-OH, polyethyleneglycols PEG_n_ and traces of water (Figures 3, 4), purified “heterogeneous” polyethoxylated (n = 3-20) lauryl/myristyl (7/3) alcohols are obtained. It was further interesting knowing the actual distribution of the hydrophobic (L/M) and hydrophilic PEO, respectively, chain homologues.

It is known [15,16] from the gas-chromatographic analysis that in the composition of LM(EO)_3_PN and LM(EO)_3_PD:

✓ the PEO chain homologues for both homologous hydrophobic series cover the interval n = 1-5, although the determined average value of the oligomerization degree, n, in the purified product is n = 3;

✓ the share of the PEO chain homologues for both homologous hydrophobic series, maximum at the beginning of the series (n = 1), decreases continuously in the series n = 1-5;

✓ the total percentage share of the PEO chain oligomers for the two L and M hydrocarbon chain homologous series is in a ratio of 2.33, similar to 7/3;

✓ lauryl and myristyl iodides, formed after the cleavage of the PEO chain with hydroiodic acid, extracted from the system and gas-chromatographically analyzed, have confirmed the L/M = 7/3 mass ratio between the hydrophobic chains.

**Figure 10:**
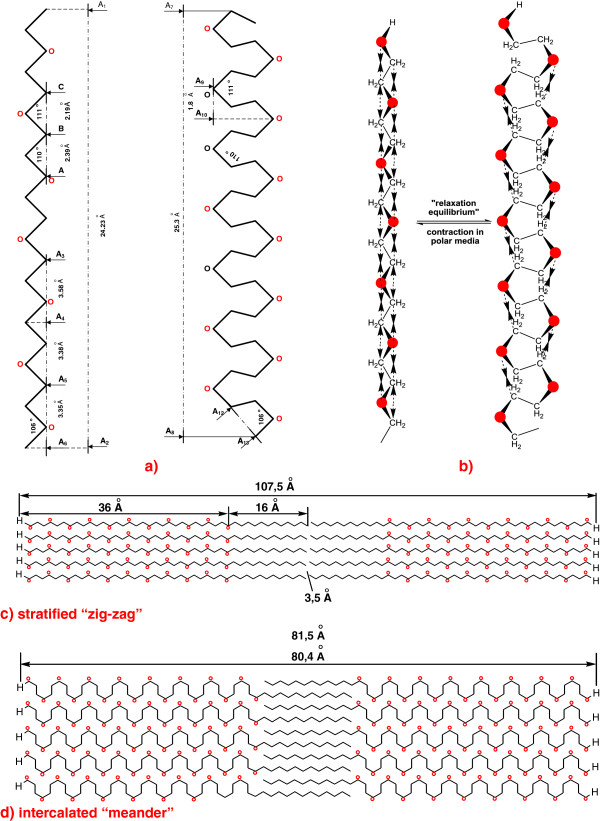
**Reaction scheme for obtaining “homogeneous” lauryl/myristyl (7/3) polyethoxylated (n = 3) alcohol LM(EO)**_**3**_**H.**

The strict nomination of each R and PEO chain homologue was performed using as standards lauryl/myristyl (7/3) alcohol LM-OH, “homogeneous” polyethoxylated (n = 3) lauryl/myristyl (7/3) alcohol LM(EO)_3_H (the adapted Williamson method) (Figure 10) and “homogeneous” β-lauryl/myristyl (7/3) polyethyleneoxy (n = 3) propionitrile, LM(EO)_3_PN [15,16].

The aforementioned standards, gas-chromatographically eluted in compliance with the same operating parameters, confirmed the same retention times with their similar homologues in purified heterogeneous LM(EO)_3_PN [15,16].

### Conformational characterization of the PEO chains. Consequences for R(EO)_n_PD

The rediscovery of crown ethers (CR) and their role as phase-transfer catalysts (PTC) (Pedersen, C., 1967) [36] represented the decisive impulse in the theoretical and practical conformational study of cyclic and acyclic PEO chains, as such and derivatized.

The striking analogy CR could not help but formulate questions and provide convincing answers about the spatial geometry of PEO chains.

The hypotheses formulated, valence angles and interatomic distances that allow free coaxial rotation and offer additional “constructive conformational details” about the “strain-free-polyoxyethylene chain”, about the modes of “packing” in a “macromolecular lattice”, specify that:

✓ the “monoclinic unit cell” contains 4 “meanders” in its structure [8-12];

✓ the “meander” contains 9 oxyethylene units (EO), (4 × 9 = 36 EO units in a monoclinic cell structure) [8,9];

✓ the “repetition interval” accepted is at 19.5 Å [13,37-40];

✓ each EO unit is “twisted” from the anterior one, so that the main PEO chain is “distorted” and returns to the initial conformation at every tenth “step” (19.5 Å) [41-44];

✓ in the “meander” conformation an EO unit has (average values) a length of 1.9 Å and diameter of 4 Å; as against the “zig-zag” conformation with the geo-metric parameters of 3.5 Å and 2.5 Å, respectively [7].

**Figure 11:**
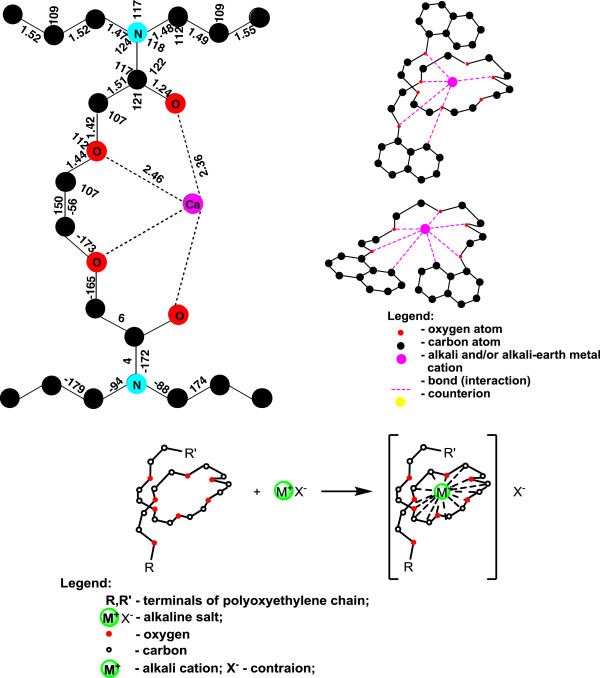
**Conformational models of polyoxyethylene (PEO) chains [**[9]**]. a**) “zig-zag” planar “meander” (Staudinger, H., 1960; Kehren, M. and colab., 1957); **b**) “zig-zag” “meander”, in contraction-relaxation equilibrium; **c**) stratified “zig-zag”; **d**) “zig-zag” “meander”, stratified and intercalated in contraction-relaxation equilibrium.

Subsequent experimental facts [42-45], Figure 11, also confirmed for the first time the approximate dimensions of the coordination “helical cavity” (ca. 6 EO units/sodium cation and ca. 7 units EO/potassium cation, respectively).

Currently it is accepted that the geometry of the “coordination cavity” is flexible, mobile, dependent on the PEO chain’s length, the geometry of the generally coordinated entities, the PEO chains being also affected by the “cooperation” or “competition” effects of the oxygen atoms in the rest of the PEO chain.

Recent literature confirms [12,14,44] (Figure 11):

✓ different behavior in solution of the PEO chains from that in unsolvated solid phase, dependent on the chain and dispersion medium;

✓ the “meander” conformation (in plane) helical (in space) is regarded as certain in solution for n ≥ 9, and in solid state for n ≥ 20;

✓ the contraction/dilatation (extension) of the polyoxyethylene PEO chains is dependent on the physico-chemical parameters employed;

✓ the geometric coordinates (diameter 4.5 Å and 2.5 Å, respectively; area 28 Å^2^ and 19 Å^2^, respectively) are flexible and conformation-dependent.

In the cases of coexisting C_12_H_25_(EO)_n_H and crown ether in equal molar concentration, each mass spectrum was dominated by the cationized C_12_H_25_(EO)_n_H rather than cationized crown ether. This may indicate that the cation affinity of helical POE is significantly higher than that of cyclic POE, although the cation selectivity of the crown ether was significantly higher than that of POE surfactants [13,14].

As the EO unit number increased, the ESI (Electrospray ionization) mass spectra could exhibit the multiply charged C_12_H_25_(EO)_n_H (average) cationized by alkali metal ions in addition to their singly cationized species [14,37,38].

Yokoyama, Y. et al. [12,14] introduced the critical EO unit numbers necessary to start producing the multiply cationized molecules, which were very dependent on the guest cation diameters as summarized in Table 3.

**Table 3:** **Critical EO unit numbers for alkali-metal ions**[12,14]

**Alkali cation**	**Double adduct**	**Triple adduct**
Li^+^	12	24
Na^+^	12	24
K^+^	15	28
Rb^+^	17	31
Ca^2+^	19	34

**Figure 12:**
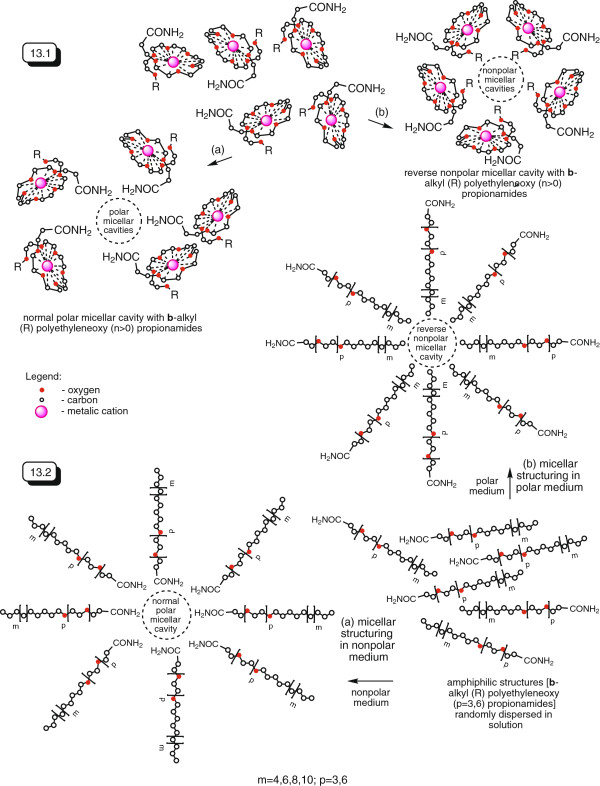
**Conformational elements, bond angles and lengths (Å), of the coordination with acyclic polyoxyethylene (PEO) chains of different sizes [**[16-20,45]**].**

Therefore, such theoretical molecular modeling simulations are very indicative of the experimental findings. Since one helical turn consisting of 6 EO residues can be recognized in the simulation, the apparent inner diameters of the horizontal section of the helical structure when including K^+^ are thought to be similar to the hole diameter of 18-crown-6 [45] (Figure 12).

Throughout our research, the obtained results have contributed decisively to the confirmation of the direct participation and the formulation of the conformational role of the PEO chains by:

✓ our own experimental observations according to which in the nucleophilic addition reactions (cyanoethylation, amidoethylation) of polyethoxylated higher alcohols purified of alcohols, polyethyleneglycols (PEG_n_) and water, the processing yields under similar conditions increase proportionally with the oligomerization degree, n, of the PEO chain [16-20];

✓ the progress reported in the literature on the knowledge of conformational performances of the PEO chains in solid, liquid (solution) state, and the various investigation methods used.

After nearly a century of investigations, similarly to other classes of macromolecular compounds, in the case of polyethyleneglycols (PEGn) and their derivatives (glyme, oligoglyme, PEGylated compounds), respectively, it can also be argued that besides a primary structure there is a secondary (conformational) and a tertiary (micellar macromolecular architectures) structure.

The main features of these spatial architectures with consequences for the study of heterogeneous β-alkyl polyethyleneoxy (n = 3-20) propionamides R(EO)_n_PD are: dimensional flexibility, transfer mobility, the existence of the “meander”, “zig-zag”, “helix” conformations with variable geometry, free coaxial rotation C-C/C-O, absence of “ring tensions” specific to rigid structures.

Recent studies [14,16,46-48] have also confirmed the effects of the oligomerization degree (n) and temperature in the 20-40°C range on the partition coefficients of “homogeneous” polyethoxylated lauryl alcohol in the series n = 2-9 at the water-isooctane interface. These investigations have led to the structuring of a database necessary for the evaluation of the same partition coefficient for heterogeneous polyethoxylated lauryl alcohol (mixture of PEO chain homologues belonging to the same structural family).

**Figure 13:**
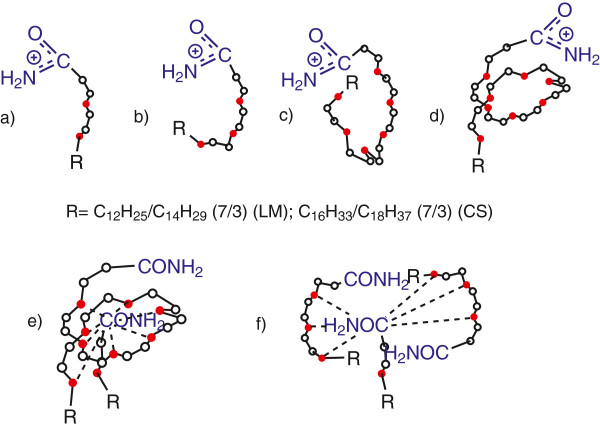
**Structure and alternative formation of normal (a) and reverse (b) micelles with the participation of R(EO)**_**n**_**PD: 13.1.** Principial schematic representation of the association/adsorption mode at the interface in a spherical micellar architecture with LM(EO)_n_PD (n ≥ 8) (helix conformation) in nonpolar (**a**) and polar (**b**) medium, respectively; 13.2. Principial schematic representation of the association/adsorption mode at the interface in a spherical micellar architecture with LM(EO)_n_PD (n = 3,6) (“zig-zag” conformation) in nonpolar (**a**) and polar medium (**b**), respectively.

The aforementioned considerations also contributed to the adapted schematic representation of the primary micellar structures of heterogeneous R(EO)_n_PD (Figures 13).

### Adsorption of heterogeneous R(EO)_n_PD (n = 3-20) at the aqueous solution-air interface. Formation of micelles

**Figure 14:**
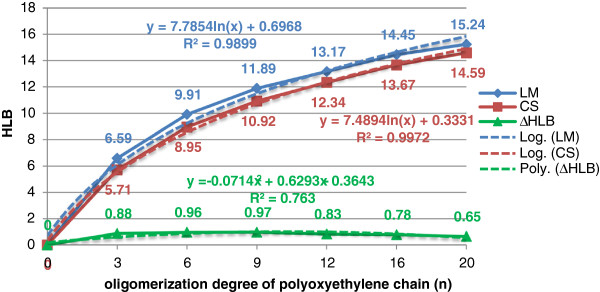
**Schematic representation of the conformation of R(EO)**_**n**_**PD: a) n = 2; b) n = 3; c) n = 5; d) n = 8 and of the conformation of the mixed architecture: e) R-O-PD/R(EO)**_**n**_**PD n = 8 (1/1) and f) R-O-PD/R(EO)**_**n**_**PD n = 3 (1/2), respectively.**

β-Alkyl polyethyleneoxy (n = 3-20) propionamides are lyophilic association colloids which group instantly in aqueous floats as macromolecular associations (lyophilic micelles) at the value corresponding to the critical micelle concentration (expressed as mol/L × 10^-5^) (Figure 14e,f).

The classic, basic colloidal characterization of heterogeneous R(EO)_n_PD does not lead to relevant interpretations.

For “homogeneous” R(EO)_n_PD, the main basic colloidal characteristics to be determined would be [34]: surface excess concentration (Γ_cmc_); surface area demand per molecule (A_min_); efficiency in surface tension reduction (p_C20_); the effectiveness of surface tension reduction (σ_cmc_); critical micelle concentration (CMC); measure of the tendency of the surfactant mentioned to adsorb at the aqueous/air interface relative to its tendency to form micelles in the bulk surfactant solution (CMC/C_20_); standard free energy of micellization (ΔG°_mic_) and β-parameter.

In the case of heterogeneous R(EO)_n_PD, the diversity of composition, structure and conformations does not allow for such an approach, but only the indicative evaluation of the following surface-active parameters: HLB balance, surface tension (σ_cmc_) and critical micelle concentration (CMC).

In the homologous series of heterogeneous hydrophobic (R) and hydrophilic (PEO) chains of R(EO)_n_PD, subsequently can also be indicatively formulated structure-surface activity correlations.

Experimental area/molecule data (A_expt_) at the air/aqueous solution interface after mixing, ideal mixing area/molecule data (A_ideal_), based upon area/molecule data at the interface before mixing, and regular solution theory have been used to explain the values of surfactant molecular interaction β parameters observed in mixed monolayers and mixed micelles [34,43].

Steric effects occur simultaneously with the dimensional change of the hydrophobic chain, and the increase of the geometric coordinates of the PEO chains with the oligomerization degree, n, reduce the interchain electrostatic repulsions. These also induce the modification of the β-parameter [34,49]. The β-parameter, β = W_AB_ - (W_AA_ + W_BB_/2)RT, evaluated for the quantification of molecular interactions of two distinct surface-active components A and B in a mixture, involves the evaluation of the mutual molecular interaction energy W at temperature T(°K). In the casuistry of heterogeneous R(EO)_n_PD is necessary the knowledge of the mutual interactions of 24 R and PEO chain homologues (Figure 2), two by two.

The critical micelle concentration, CMC, was determined by surface tension techniques. In the case of surface tension measurements, the CMC values are taken as the molar concentrations at the intersection of the two linear parts of the relationship γ = f(logC), above and below the discontinuity.

Corroborating the aforementioned conformational data of PEO in the literature with the structure of various R(EO)_n_PD, in Figure 13 are presented several concrete instances of association at the hydrophobic or hydrophilic separation interface (polar and nonpolar media) of R(EO)_n_PD for n = 3 and 6. Micellar architectures with polar and nonpolar cavities are thus structured as “solubilization”, sequestration and transfer spaces of potential entities in the sanitation practice.

In view of the participation of β-substituted higher aliphatic propionamides as cosurfactants in sanitation recipes, the simultaneous or alternative presence of normal and reverse micelles must be accepted as a very probable reality (Figure 13).

### Adsorption of heterogeneous R(EO)_n_PD homologues mixtures at the aqueous solution-air interface. Formation of mixed micelles

Mixtures of two or more different homologues in the R(EO)_n_PD series show positive or negative “synergistic” interdependences [34,49]. The overall interfacial properties of the mixture of homologues can be more pronounced than those of a “homogeneous” (unitary) R(EO)_n_PD structure. Consequently in many technological applications mixtures of various surface-active structures are preferred and the interactions between them afford an understanding of the role of each individual structure and make possible their selection in a rational systematic manner for the optimization of the colloidal properties.

The synergisms or antagonisms of a series of structural homologues of R(EO)_n_PD and the relationship with the fundamental colloidal characteristics, that is the role of cosurfactants, represents an important area and one of the objectives of this work. The indicative conclusions of such a study in perspective can substantiate the selection of the R(EO)_n_PD unitary structures in the structuring of surface-active couples that would ensure optimized applicative interfacial properties (Figure 14).

β-Alkyl (R) oxy propionamides R-O-PD can be considered “parent compound” or “parent compound” products for the homologous series of R(EO)_n_PD (n = 3-20), since the only “ether bridge” newly created through nucleophilic addition does not provide water solubility. The interest for knowing the possibility of integrating R-O-PD (with no surface active properties) and the rest of the homologous series in different sanitation systems (solubilization) becomes evident if it is admitted the reality that polyethoxylated (n = 3-20) higher aliphatic β-propionamides, as technical products, inevitably contain at the beginning of the homologous series (n = 3-6) these structures that affect the individual colloidal characteristics. Knowing on the other hand the conformational properties of the PEO chains (Figure 14), the remarkable possibility of mutual interaction (“sequestration” and micellar solubilization), we proposed the association of R-O-PD (n = 0)/R(EO)_n_PD (n = 12) in the (1/1) molar ratio, followed by the evaluation of the indicative cumulative colloidal characteristics. The experimental values obtained were interpreted through mixed micellar associations with positive synergistic effects, capable of cumulated surface activity (Figure 14). In many surface-active recipes in technological practice, mixtures of surfactant compounds dominate the global colloidal behavior relative to the individual colloidal characteristics of the participants due to certain resulting effects. Also in many cases commercial technical aspects (inhomogeneous raw materials, side products of processing or unreacted products) define the overall surface-active behavior. The mutual molecular interactions provide with high probability the associated colloidal systems (mixed) with the perspective of an optimized colloidal behavior [34].

Most R(EO)_n_PD (n = 3-20) studied (Table 1) are water-soluble surface-active compounds with the exception of the homologue (n = 0).

In this work the corresponding values of surface tension and critical micelle concentration, respectively, for β-alkyl C_12_H_25_/C_14_H_29_ (7/3); C_16_H_33_/C_18_H_37_ (7/3) oxy (n = 0) propionamide shall refer to the associated system 1/1 of β-propionamide (n = 0) with β-propionamide (n = 12) (water-soluble). The mixed micelle (n = 0)/(n = 12) allows for surfactant activity cumulated with overall positive synergistic effects (Figure 14).

### The hydrophilic/hydrophobic balance in the homologous series of heterogeneous R(EO)_n_PD. Structural correlations

The cosurfactant quality of R(EO)_n_PD in binary or ternary surfactant system, designed for sanitation by the CIP (“clean in place”) process, can also be appreciated semiempirically with the help of the HLB scale. In the case of the structures containing PEO chains as the dominant hydrophilic group, the contribution of the amide function can be insignificant, and the simplified GRIFFIN relationship HLB = E/5 (where E is the percentage by mass of ethene oxide (EO) in the surface-active structure) can offer sufficient elements for the proposed characterization.

Thus one can predict the role of wetting agents, cleaning agents and of micellar solubilization of dirt present on contact interfaces throughout sanitation.

**Figure 15:**
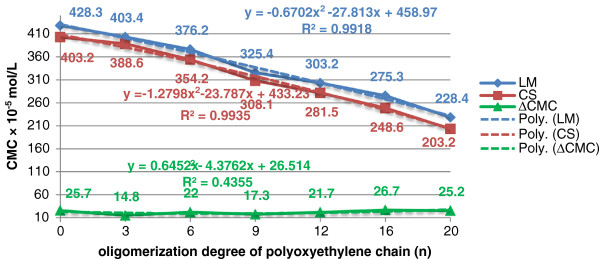
**Dependence of the HLB balance on the oligomerization degree (n) of the polyoxyethylene chain in the homologous series of LM(EO)**_**n**_**PD and CS(EO)**_**n**_**PD, respectively.**

The introduction of polyoxyethylene chains with variable (n = 3-20) oligomerization degree in the series of β-substituted aliphatic propionamides changes controllingly, gradually, the hydrophilic/hydrophobic index (HLB) (Figure 15). The dependence after a logarithmic relationship suggests through the correlation coefficient R^2^>0.98 a high similarity to reality. Two aspects are additionally noted: a difference ΔHLB_average_ = 0.845 between the two series of β-substituted aliphatic propionamides LM and CS and a range of HLB values covering the interval 5.71-15.24, specific to all colloidal competences (dispersants, emulsifiers, wetting agents, sequestrants, foaming agents, defoamers, co-surfactants etc.) (Table 4).

**Table 4:** **Correlation of HLB with the average oligomerization degree (n) and the scope in the homologous series of heterogeneous R(EO)**_**n**_**PD**[2,3]

**n**	**HLB**	**Scope of heterogeneous R(EO)**_**n**_**PD**
3	6	Emulsifier W/O
6	8-10	Wetting agent
9	10-12	Emulsifier O/W
12	12-14	Detergent, emulsifier O/W
16	13-15	Detergent
20	15-16	Micellar solubilization agent

The HLB balance in the series of the structure homologues of heterogeneous R(EO)_n_PD will be strongly influenced by the evolution of the lyophilic-lyophobic equilibrium inside it. Increasing the average oligomerization degree (n) of the PEO chain increases the hydrophilicity and shifts the indicative HLB values towards the upper end of the variation range, after a logarithmic mathematical relationship (Table 4) (Figure 15).

### The critical micelle concentration in the homologous series of “heterogeneous” R(EO)_n_PD. Structural correlations

**Figure 16:**
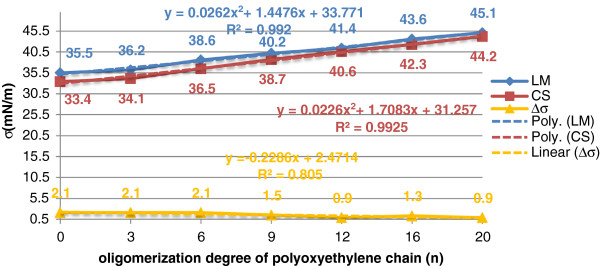
**Dependence of critical micelle concentration expressed as mol/L** **×** **10**^**-5 **^**on the oligomerization degree (n) of the polyoxyethylene chain in the homologous series of LM(EO)**_**n**_**PD and CS(EO)**_**n**_**PD, respectively.**

In the same homologous series of β-alkyl polyethyleneoxy substituted aliphatic propionamides the critical micelle concentration decreases simultaneously with increasing the oligomerization degree (n) of the polyoxyethylene chains, after a polynomial mathematical relationship with the correlation coefficient R^2^>0.99 (Figure 16). In this case, too, the experimental values of CCM for the series with the LM hydrophobic chain are higher by ΔCCM_average_ = 21.91 mol/L × 10^-5^ from those of the series with the CS hydrophobic chain, which leads us to the conclusion that over the entire range of the homologous series, for a certain value of CCM of the CS series at the same oligomerization degree (n) of the polyoxyethylene chain, the CCM value of the LM series is higher on average by 21.91 mol/L × 10^-5^ than for the first series.

Equating the molar concentration in mass units (g/L) for the representatives of the two homologous series, we find that:

✓ the CCM value (g/L) decreases by 0.81 g/L in the series of polyoxyethylene chain homologues (n = 3-20) for LM and by 1.08 g/L for CS, respectively;

✓ for the same oligomerization degree (n), the CCM value (g/L) is higher for the LM compared to the CS series.

Interpreting the two previous statements, is confirmed that the efficiency of surface tension reduction for the homologous series CS is superior to the homologous series LM.

### The surface tension in the homologous series of heterogeneous R(EO)_n_PD. Structural correlations

Surface tension is dependent on the structural characteristics of the studied heterogeneous LM(EO)_n_PD and CS(EO)_n_PD, respectively. Since the interface equilibrium must be established in a short time, in the sanitation practice and washing floats the phenomenon acquires a special practical significance. Two aspects are required to be reviewed separately: the capacity of surface tension reduction, the concentration of β-alkyl polyethyleneoxy (n = 3-20) propionamides necessary to achieve a certain effect of surface tension reduction, and the efficiency of surface tension reduction expressed by the minimum value at which β-alkyl polyethyleneoxy (n = 3-20) propionamides are able to reduce the surface tension.

Assimilating heterogeneous R(EO)_n_PD into the group of “hybrid” ionic-nonionic surface-active compounds, it can be stated with sufficient accuracy that they will present the sum of individual colloidal properties of higher aliphatic amides (the ionic part) and of polyethoxylated higher alcohols (the nonionic part). The capacity and effectiveness of surface tension reduction in aqueous solution of R(EO)_n_PD generally evolves inversely proportional to the modification of the size of the hydrophilic PEO chain, the structure (unsaturated, branched) of the hydrophobic chain, the movement of the ionized hydrophilic group from the middle to end of the R chain, the intensity of the mutual electrostatic repulsion [34]. The transfer of an R and PEO chain homologue from the solution to the air-separation interface depends on the size, mobility and electrical charge (and mutual repulsion, respectively) between them.

**Figure 17:**
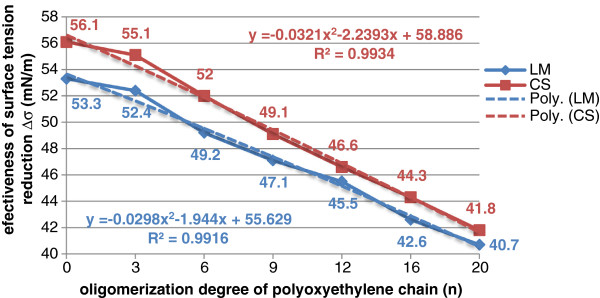
**Dependence of surface tension (mN/m) on the oligomerization degree (n) of the polyoxyethylene chain in the homologous series of LM(EO)**_**n**_**PD and CS(EO)**_**n**_**PD, respectively.**

**Figure 18:**
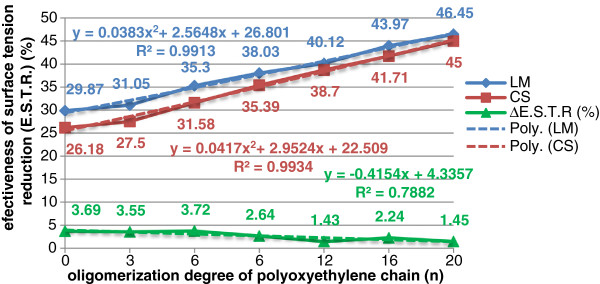
**Variation of the effectiveness of surface tension reduction against the oligomerization degree (n) of the polyoxyethylene chain in the homologous series of LM(EO)**_**n**_**PD and CS(EO)**_**n**_**PD, respectively.**

In the surface-active structures of heterogeneous R(EO)_n_PD evaluated (Figure 17), increasing the oligomerization degree of the PEO chain (n > 6) induces an increased capacity of surface tension reduction, simultaneously with decreasing the effectiveness of adsorption at the water/air interface (Figure 18). The phenomenon is justified by the unfavorable steric effects resulting from the conformational characteristics of the hydrophilic PEO chains for n > 6, the difficulties of “packing” at the interface and, last but not least, the intensification of the electrostatic repulsions. Scenarios of this type diversify in the case of mixtures of different R and PEO chain homologues (Figure 14).

In the homologous series of β-substituted higher aliphatic propionamides, the surface tension increases simultaneously with the oligomerization degree (n) of the polyoxyethylene chains after a polynomial mathematical relationship with correlation coefficients R^2^>0.99 (Figure 17). The experimental values reported for the LM series are higher by Δσ_average_ = 1.558 mN/m than those of the CS series, which leads also to the conclusion the effectiveness of surface tension reduction in the homologous CS series is superior to that of the LM series (Figure 17).

It can also be noted that for the same oligomerization degree (n) of the polyoxyethylene chain, the effectiveness of surface tension reduction is greater for the lower hydrophobic (hydrocarbon) chains than for the higher ones.

In the homologous series of heterogeneous R(EO)_n_PD, the capacity of adsorption at the water/air separation interface tends to increase due to the hydrophilic weakly ionic polar primary amide group (Figure 13) for same hydrophobic chain R. The electrostatic repulsions due to the structure are low and favor the degree of “packing” at the interface.

Increasing the oligomerization degree (n) in a homologous series is proportional to ethene oxide content, and therefore with the hydrophilicity and solubility of the structure. That is why β-substituted aliphatic propionamides (n = 3), with lower solubility than the higher homologues of the series (n = 20), have a more pronounced hydrophobic character, therefore higher tendency of orientation at the separation interface (e.g., water/air) and effectiveness of surface tension reduction. The situation reverses for the homologues with (n = 20) (Figure 18). The same explanation is valid for the argumentation of the difference in effectiveness between the LM and CS series.

**Figure 19:**
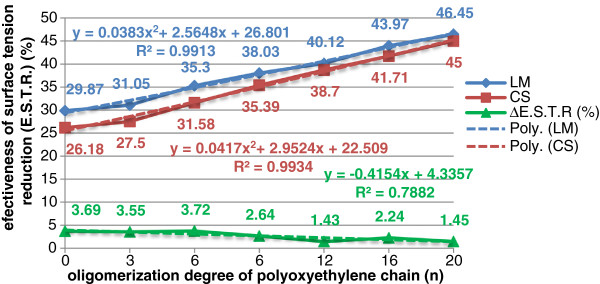
**Variation of the effectiveness of surface tension reduction for a certain oligomerization degree (n) of the polyoxyethylene chain against the surface tension of pure water in and between the homologous series of LM(EO)**_**n**_**PD and CS(EO)**_**n**_**PD, respectively.**

The interpretations made earlier about the effectiveness of surface tension reduction in the homologous series of β-substituted aliphatic propionamides studied can also be quantified if we compare the recorded experimental values to the value of the surface tension of double distilled water for distilled water (Figure 19).

Preliminary indicative evaluations of the parameter pC_20_ for heterogeneous R(EO)_n_PD, the negative logarithm of concentration of heterogeneous R(EO)_n_PD in the bulk phase required to produce a 20 mN/m (dyn/cm) reduction in the surface tension of the solvent [34,50], led to inconclusive values with wide dispersion, without the possibility of even approximate correlation and of formulation of evolution trends. Attempting to explain the phenomenon, we resorted to liquid-chromatographic determination of the distribution of the hydrophilic PEO chain homologues for n > 6. It was found that the statistical distribution of the PEO chain homologues no longer presents a symmetry detected for n < 6, but instead the shift of the major share of the PEO homologues towards values much larger than the average value determined of the oligomerization degree (Δn = 2-3 EO units) [15-17].

These confirm the role of the homogeneous (unitary) character of R(EO)_n_PD in the evaluation of the basic colloidal properties.

## Materials and methods

### Materials

1. heterogeneous β-alkyl (C_12_H_25_/C_14_H_29_) (7/3) polyethyleneoxy (n = 0-20) propionamides [LM(EO)_n_PD] (mixture of hydrophobic R chain homologues in a mass ratio (7/3) and hydrophilic polyoxyethylene PEO chain homologues (n = 0-20), respectively) (Department of Food Technologies, Faculty of Food Processing Technology, Banat’s University of Agricultural Sciences and Veterinary Medicine of Timişoara, Romania) [6];

2. heterogeneous β-alkyl (C_16_H_33_/C_18_H_37_) (7/3) polyethyleneoxy (n = 0-20) propionamides [CS(EO)_n_PD] (mixture of hydrophobic R chain homologues in a mass ratio (7/3) and hydrophilic polyoxyethylene PEO chain homologues (n = 0-20), respectively) (Department of Food Technologies, Faculty of Food Processing Technology, Banat’s University of Agricultural Sciences and Veterinary Medicine of Timişoara, Romania) [6];

3. polyethoxylated (n = 3-20) lauryl/myristyl (7/3) alcohols [LM(EO)_n_H] (technical products), purified of free lauryl/myristyl (7/3) alcohol, polyethyleneglycols (PEG_n_) and traces of water (mixture of hydrophobic R chain homologues in a mass ratio (7/3) and hydrophilic polyoxyethylene PEO chain homologues (n = 0-20), respectively) (S.C. Romtensid S.A. and Department of Food Technologies, Faculty of Food Processing Technology, Banat’s University of Agricultural Sciences and Veterinary Medicine of Timişoara, Romania) [6,8,15] (Table 1);

4. polyethoxylated (n = 3-20) cetyl/stearyl (7/3) alcohols [CS(EO)_n_H] (technical products) purified of free cetyl/stearyl (7/3) alcohol, polyethyleneglycols (PEG_n_) and traces of (mixture of hydrophobic R chain homologues in a mass ratio (7/3) and hydrophilic polyoxyethylene PEO chain homologues (n = 0-20), respectively) (S.C. Romtensid S.A. and Department of Food Technologies, Faculty of Food Processing Technology, Banat’s University of Agricultural Sciences and Veterinary Medicine of Timişoara, Romania) [6,8,15] (Table 1);

5. “homogeneous” polyethoxylated (n = 3) lauryl/myristyl (7/3) alcohol [LM(EO)_3_H] (Department of Food Technologies, Faculty of Food Processing Technology, Banat’s University of Agricultural Sciences and Veterinary Medicine of Timişoara, Romania) [17,18];

6. “homogeneous” β-lauryl/myristyl (7/3) polyethyleneoxy (n = 3) propionitrile [LM(EO)_3_PN] (Department of Food Technologies, Faculty of Food Processing Technology, Banat’s University of Agricultural Sciences and Veterinary Medicine of Timişoara, Romania) [17,18];

7. lauryl/myristyl alcohol (LM-OH), mass ratio (7/3) (S.C. Romtensid S.A./Alfol 1214 Condea-Germany) (Table 1);

8. cetyl/stearyl alcohol (CS-OH), mass ratio (7/3) (S.C. Romtensid S.A./Alfol 1618 Condea-Germany) (Table 1);

9. N,N,N-trimethyl-N-β-lauryl/myristyl (7/3) oxy-ethylammonium chloride (LM-O-EC-1.1.1.) [15,16];

Reagents (Sigma-Aldrich, Merck): acrylamide; organic solvents; acrylonitrile (inhibited with 35–45 ppm hydroquinone monomethyl ether); triethylene glycol; p-toluenesulfonyl chloride

### Methods

1. Determination of the cloud point of nonionic surface-active agents obtained by condensation of ethylene oxide [51];

2. Determination of surface tension. Ring variant [52];

Air/water solution surface tension were measured continuously using a Krüss K20S automatic tensiometer (Germany), equipped with a duNuoy Pt-Ir ring (resolution ±0.01 mN · m^-1^) at 25 ± 0.1°C. Sets of experiments were taken at intervals until no significant change occurred in the tension [48]. Equilibrium surface tension data were reproducible within 0.2 mN · m^-1^.

The CMC values of heterogeneous R(EO)_n_PD (mol/L · 10^-5^) were determined by surface tension techniques. In this case of measurements the CMC values are taken as the molar concentration at the intersection of the two linear parts of the relationship σ = f(logC) above and below the discontinuity [48]. Initial concentration was 1 mol/L · 10^-2^.

Heterogeneous R(EO)_n_PD were purified above 99%, the purity was determined by titration in nonaqueous dipolar aprotic medium (DMSO, DMF) with HClO_4_ 0.1 N [53]. The average molecular weight (M_av_) was evaluated with the relationship: M_av_ = M_R_ + n · 44 + MC_3_H_6_NO, where M_R_ represents the average molecular weight of the hydrophobic chains LM C_12_H_25_/C_14_H_29_ (7/3) and CS C_16_H_33_/C_18_H_37_ (7/3), respectively, determined by gas chromatography [15,16], and n the average oligomerization degree of the hydrophilic polyoxyethylene PEO chain, determined iodometrically [35].

3. Polyethoxylated derivatives. Iodometric determination of oxyethylene groups [35].

4. Preparation of “homogeneous” polyethoxylated lauryl/myristyl (7/3) alcohol LM(EO)_3_H. Is performed by following the reaction scheme in Figure 10[15,16].

### Equipment

Digital tensiometer Krüss K20S.

## Conclusions

Heterogeneous β-alkyl C_12_H_25_/C_14_H_29_ (7/3) and C_16_H_33_/C_18_H_37_ (7/3), respectively, polyethyleneoxy (n = 3-20) propionamides R(EO)_n_PD, nonionic surface-active structures, accessible under mild processing conditions, offer a wide range of colloidal properties by the controlled directing of the hydrophilic/hydrophobic share in their structure.

The synergistic cumulation in the same structural architecture of polyoxyethylene chains with various oligomerization degrees (n = 3, 6, 9, 12, 16, 20) (average values of the statistical distribution) was confirmed for the first time to be a realistic method of optimizing the initial limited solubility of classic higher amides with the exception of R-O-PD, “parent compound of the homologous series”.

Although falling into a limited “niche” of the surface-active spectrum through composition, structure, methodology of production, heterogeneous R(EO)_n_PD have strong connections with other areas of general interest of knowledge (supramolecular chemistry, coordinative chemistry, PEGylation, conformational chemistry etc.).

The heterogeneous R(EO)_n_PD studied constitute a new “hybrid” domain in the category of surface-active compounds, with three defining characteristics which mark decisively their basic colloidal properties:

✓ the heterogeneous character (series of homologues), due to the dispersion of the oligomerization degree of the hydrophilic PEO chains and determinant hydrophobic R chain;

✓ the particular conformational characteristics of the PEO chains, depending on the average oligomerization degree, n;

✓ the possible and very probable presence of side products, specific to the industrial synthesis of polyethoxylated higher alcohols, (free higher alcohols, polyethyleneglycols, traces of water).

The differences between the “homogeneous” and “heterogeneous” character of R(EO)_n_PD have also led to limiting the possibilities for a colloidal classic investigation compared to a proper unitary surface-active structure.

The heterogeneous R(EO)_n_PD studied were obtained from polyethoxylated higher alcohols LM(EO)_n_H, R and PEO chain homologues, after purification of free higher alcohols, polyethyleneglycols and traces of water. The operation was performed by repeated liquid/liquid extractions in various solvent systems: ethyl acetate/saturated NaCl solution/chloroform; 96% ethyl alcohol/petroleum ether.

The presence of these structural and compositional components defined the heterogeneous character of R(EO)_n_PD. This was confirmed through the comparative gas-chromatographic analysis of heterogeneous LM(EO)_n_PN [β-lauryl/myristyl (7/3) polyethyleneoxy (n = 3) propionitrile] with “homogeneous” LM(EO)_3_PN (reference standard) obtained by the adapted Williamson synthesis.

This approach allowed the evaluation of the basic colloidal properties indicatively (hydrophilic/hydrophobic balance, surface tension, critical micelle concentration). The comparative integrated evaluation of the literature data on the conformational behavior of PEO chains and the extended electronic system in the polar hydrophilic amide group, allowed the formulation of mechanisms for interaction and micellar association in the homologous series of R(EO)_n_PD, able to justify their colloidal manifestations. The usefulness of complex scientific efforts towards heterogeneous surface-active systems argue towards accessing R(EO)_n_PD as co-surfactants along with nonionic soaps, alkaline or ammonium β-alkyl (R) polyethyleneoxy (n = 3-20) propionates, [R(EO)_n_PC^-^M^+^], in sanitation recipes.

The indicative colloidal evaluations presented allowed the formulation of informative structure-surface-active characteristics correlations and mathematical processing with correlation coefficients R^2^ ≥ 0,58.

The working premises formulated initially, the proposed major objectives of the study, together with the work strategies applied, will broaden interest in diversifying the category of ionic-nonionic surface-active compounds, but also for further research to complete the expressed hypotheses.

## Competing interests

The authors declare that they have no competing interests.

## Authors’ contributions

TT mathematically processed and interpreted the experimental data. A.R. evaluated the basic surface-active competences in the homologous series of substituted β-alkyl (C_12_/C_18_) polyethyleneoxy (n = 0-20) propionamides studied. GB obtained and characterized chemically the β-substituted higher aliphatic propionamides necessary for the study. All authors read and approved the final manuscript.
